# Diagnostic accuracy of magnetic resonance enterography and small bowel ultrasound for the extent and activity of newly diagnosed and relapsed Crohn's disease (METRIC): a multicentre trial

**DOI:** 10.1016/S2468-1253(18)30161-4

**Published:** 2018-06-18

**Authors:** Stuart A Taylor, Susan Mallett, Gauraang Bhatnagar, Rachel Baldwin-Cleland, Stuart Bloom, Arun Gupta, Peter J Hamlin, Ailsa L Hart, Antony Higginson, Ilan Jacobs, Sara McCartney, Anne Miles, Charles D Murray, Andrew A Plumb, Richard C Pollok, Shonit Punwani, Laura Quinn, Manuel Rodriguez-Justo, Zainib Shabir, Andrew Slater, Damian Tolan, Simon Travis, Alastair Windsor, Peter Wylie, Ian Zealley, Steve Halligan, METRIC study investigators, Jade Dyer, Pranitha Veeramalla, Sue Tebbs, Steve Hibbert, Richard Ellis, Fergus Thursby-Pelham, Richard Beable, Nicola Gibbons, Claire Ward, Anthony O'Connor, Hannah Lambie, Rachel Hyland, Nigel Scott, Roger Lapham, Doris Quartey, Deborah Scrimshaw, Helen Bungay, Maggie Betts, Simona Fourie, Niall Power, Rajapandian Ilangovan, Uday Patel, Evgenia Mainta, Phillip Lung, Ian Johnston, Mani Naghibi, Morgan Moorghen, Adriana Martinez, Francois Porte, Christopher Alexakis, James Pilcher, Anisur Rahman, Jonny Vlahos, Rebecca Greenhalgh, Anita Wale, Teresita Beeston, Wivijin Piga, Joey Clemente, Farooq Rahman, Simona de Caro, Shameer Mehta, Roser Vega, Roman Jastrub, Harbir Sidhu, Hameed Rafiee, Mairead Tennent, Caron Innes, Craig Mowat, Gillian Duncan, Steve Morris

**Affiliations:** a Centre for Medical Imaging, University College London (UCL), London, UK; b Institute of Applied Health Research, National Institute of Health and Research Birmingham Biomedical Research Centre, College of Medical and Dental Sciences, University of Birmingham, Birmingham, UK; c Intestinal Imaging Centre, St Mark's Hospital, London North West University Healthcare (LNWUH) National Health Service (NHS) Trust, Harrow, UK; d Department of Gastroenterology, University College Hospital, London, UK; e Department of Gastroenterology, St James's University Hospital, Leeds Teaching Hospitals NHS Trust, Leeds, UK; f Inflammatory Bowel Disease Unit, St Mark's Hospital, LNWUH NHS Trust, Harrow, UK; g Department of Radiology, Portsmouth Hospitals NHS Trust, Portsmouth, UK; h Centre for Medical Imaging, UCL, London, UK; i Department of Psychological Sciences, Birkbeck University of London, London, UK; j Department of Gastroenterology and Endoscopy, Royal Free London NHS Foundation Trust, London, UK; k Department of Gastroenterology, St George's Hospital, London, UK; l Department of Histopathology, University College Hospital, London, UK; m Comprehensive Clinical Trials Unit at UCL, Institute of Clinical Trials and Methodology, Holborn, London, UK; n Department of Radiology, Oxford University Hospitals NHS Trust, Oxford, UK; o Department of Radiology, St James's University Hospital, Leeds Teaching Hospitals NHS Trust, Leeds, UK; p Translational Gastroenterology Unit, Oxford University Hospitals, Oxford, UK; q Department of Surgery, University College Hospital, London, UK; r Department of Radiology, Royal Free London NHS Foundation Trust, London, UK; s Department of Radiology, Ninewells Hospital, Dundee, UK

## Abstract

**Background:**

Magnetic resonance enterography (MRE) and ultrasound are used to image Crohn's disease, but their comparative accuracy for assessing disease extent and activity is not known with certainty. Therefore, we did a multicentre trial to address this issue.

**Methods:**

We recruited patients from eight UK hospitals. Eligible patients were 16 years or older, with newly diagnosed Crohn's disease or with established disease and suspected relapse. Consecutive patients had MRE and ultrasound in addition to standard investigations. Discrepancy between MRE and ultrasound for the presence of small bowel disease triggered an additional investigation, if not already available. The primary outcome was difference in per-patient sensitivity for small bowel disease extent (correct identification and segmental localisation) against a construct reference standard (panel diagnosis). This trial is registered with the International Standard Randomised Controlled Trial, number ISRCTN03982913, and has been completed.

**Findings:**

284 patients completed the trial (133 in the newly diagnosed group, 151 in the relapse group). Based on the reference standard, 233 (82%) patients had small bowel Crohn's disease. The sensitivity of MRE for small bowel disease extent (80% [95% CI 72–86]) and presence (97% [91–99]) were significantly greater than that of ultrasound (70% [62–78] for disease extent, 92% [84–96] for disease presence); a 10% (95% CI 1–18; p=0·027) difference for extent, and 5% (1–9; p=0·025) difference for presence. The specificity of MRE for small bowel disease extent (95% [85–98]) was significantly greater than that of ultrasound (81% [64–91]); a difference of 14% (1–27; p=0·039). The specificity for small bowel disease presence was 96% (95% CI 86–99) with MRE and 84% (65–94) with ultrasound (difference 12% [0–25]; p=0·054). There were no serious adverse events.

**Interpretation:**

Both MRE and ultrasound have high sensitivity for detecting small bowel disease presence and both are valid first-line investigations, and viable alternatives to ileocolonoscopy. However, in a national health service setting, MRE is generally the preferred radiological investigation when available because its sensitivity and specificity exceed ultrasound significantly.

**Funding:**

National Institute of Health and Research Health Technology Assessment.

## Introduction

Small bowel imaging is fundamental for comprehensive phenotyping of Crohn's disease and essential to direct therapeutic strategy.^1^ Barium fluoroscopy has long been the bedrock of small bowel investigation, providing detailed mucosal assessment.^2^ However, in the past 5–10 years enthusiasm has dwindled, and barium fluoroscopy is being increasingly replaced by cross-sectional imaging, namely computed tomography enterography (CTE), magnetic resonance enterography (MRE), and ultrasound. Advocates of cross-sectional imaging stress that these techniques assess the bowel wall and beyond, complementing endoscopic visualisation. As barium fluoroscopy is abandoned, dissemination of the various cross-sectional imaging technologies has been relatively uncontrolled, despite a paucity of supportive data from methodologically sound prospective multicentre studies. This scarcity of robust evidence is concerning given the pivotal role assumed by small bowel imaging over the lifetime of patients with Crohn's disease.

Of the available modalities, MRE and ultrasound are preferred^3^ since they avoid irradiating generally young patients who require repeat imaging.^4^ Enteric ultrasound is longer established,^5^ requires little patient preparation, and the technology is widely available. However, questions remain over accuracy, particularly in the proximal bowel and deep pelvis,^6^ and perceived interobserver variability.^7^ Conversely, MRE is a newer innovation,^8^ requires oral contrast and access to advanced technology imaging platforms, which are comparatively restricted in many health-care settings.

**Research in context**

**Evidence before this study**

Cross-sectional imaging is fundamental for diagnosis and management of Crohn's disease and is replacing barium fluoroscopic techniques, which have been the bedrock of small bowel imaging for many years. However, dissemination of cross-sectional imaging has occurred despite a paucity of supportive data from prospective multicentre studies recruiting consecutive and unselected patients. Emphasis is placed on magnetic resonance enterography (MRE) and enteric ultrasound because they avoid ionising radiation. Clinical uptake of ultrasound has been hampered by concerns over diagnostic accuracy and perceived high levels of interobserver variation. MRE is a newer innovation that necessitates access to comparatively restricted advanced technology imaging platforms. We searched PubMed and Embase in January, 2018, for articles published between Jan 1, 1990, and Jan 1, 2018, without language restriction. We used MeSH and full-text search for “Crohn's disease”, “magnetic resonance imaging”, “ultrasound”, and “diagnostic accuracy”. We retrieved primary literature but we placed emphasis on meta-analyses and systematic reviews using appropriate filters. We found several meta-analyses, which generally suggest that MRE and ultrasound have similar sensitivity for detection and activity assessment of small bowel Crohn's disease. However, the primary literature has limitations. Most studies are small, single-centre explanatory trials, recruiting fewer than 50 patients. Tests are rarely compared in the same patients, introducing bias caused by differences between patients and disease phenotype, and use inconsistent reference standards. For example, in one meta-analysis, just one of 33 included studies compared MRE and ultrasound directly in the same patients. Many studies also rate poorly on the Quality Assessment of Diagnostic Accuracy Studies tool.

**Added value of this study**

To our knowledge, this study is the largest prospective multicentre trial to date comparing the diagnostic accuracy of MRE and ultrasound for the presence, extent, and activity of enteric Crohn's disease, with the use of a construct reference standard incorporating 6 months of patient follow-up. We used a pragmatic trial design to better assess test performance in routine clinical practice, and we used the preferred method for diagnostic accuracy studies by comparing tests in the same patients. Both tests achieved high accuracy for detecting and localising small bowel Crohn's disease, but sensitivity and specificity for small bowel disease presence and extent were significantly greater for MRE than for ultrasound.

**Implications of all the available evidence**

Both ultrasound and MRE achieve high diagnostic accuracy for the extent and activity of small bowel Crohn's disease in newly diagnosed patients and those who have relapsed. Although both tests are valid first-line investigations, MRE is generally the preferred radiological investigation when available because its sensitivity and specificity exceed ultrasound significantly when tested in a prospective multicentre trial setting. Future research should investigate the role of cross-sectional imaging in patients with non-specific abdominal symptoms without an established diagnosis of Crohn's disease, and the complementary role of MRE and ultrasound in targeted follow-up of patients with Crohn's disease with an established disease phenotype.

Although meta-analyses^6^, ^9^, ^10^, ^11^, ^12^, ^13^, ^14^, ^15^, ^16^, ^17^, ^18^, ^19^, ^20^ suggest that MRE and ultrasound have similar accuracy for diagnosing and staging Crohn's disease, the primary literature is of questionable quality. Most studies^17^, ^20^, ^21^ are small and done in a single centre, and few compare tests directly in the same patients, despite this being advocated as an optimal method for diagnostic accuracy studies.^22^ For example, in their meta-analysis,^15^ Greenup and colleagues found that just one of 33 included studies compared MRE and ultrasound directly in the same patients. Additionally, very few studies use a construct reference standard model (panel diagnosis), which incorporates concepts of diagnostic test validation based on patient outcomes and has distinct methodological advantages when a single reference standard is elusive.^23^

To redress this, we did a multicentre trial to elucidate and then directly compare the diagnostic accuracy of MRE and ultrasound for small bowel Crohn's disease against a construct reference standard incorporating patient follow-up. To reflect normal clinical practice, we recruited both newly diagnosed patients and those with established disease in whom luminal relapse was suspected.

## Methods

### Study design and participants

The METRIC study is a multicentre trial that compares the diagnostic accuracy of MRE and enteric ultrasound for the presence, extent, and activity of small bowel Crohn's disease in newly diagnosed patients or patients with established disease and suspected relapse. We achieved ethics committee approval in September, 2013 (13/SC/0394). The trial was supervised by University College London's Comprehensive Clinical Trials Unit and overseen by independent Data Monitoring and Trial Steering Committees. All patients recruited gave written informed consent. The full trial protocol has been published,^24^ and can be found online.

We recruited patients from eight UK National Health Service (NHS) teaching and general hospitals, representative of institutions likely to implement MRE and ultrasound for patient management (appendix p 1). All sites had an established inflammatory bowel disease service and were already doing MRE and ultrasound as part of usual clinical practice.

Patients were eligible for the newly diagnosed group if they had been diagnosed with Crohn's disease in the 3 months preceding recruitment on the basis of conventional diagnostic criteria, or when Crohn's disease was strongly suspected on the basis of imaging or endoscopic features but pending final diagnosis. Eligible patients had already had colonoscopy or were awaiting it at recruitment. Patients in whom the final diagnosis was not Crohn's disease were subsequently excluded.

Patients were eligible for the suspected luminal relapse group if they had established Crohn's disease (>3 months) and there was a strong clinical suspicion of luminal relapse based on either objective markers of inflammatory activity (C-reactive protein [CRP] concentration >8 mg/L or faecal calprotectin concentration >100 μg/g), symptoms suggestive of luminal stenosis (including obstructive symptoms, such as colicky abdominal pain, vomiting), or abnormal endoscopy. Eligible patients for both groups were aged 16 years or older. Patients were ineligible if they were pregnant or if they had contraindications to MRI. Those with psychiatric or other disorders who were unable to give informed consent were also excluded, as were those with evidence of severe or uncontrolled systemic disease. Patients in the newly diagnosed group were excluded if they had surgical resection before colonoscopy.

Members of the local research team identified suitable patients from outpatient clinics, multidisciplinary team meetings, and inpatient wards, and they took informed consent from consecutive, unselected, eligible patients. A screening log detailed all approached patients and reasons for non-participation, if applicable. We collated patient demographics and clinical data (eg, age, sex, Montreal classification [relapse group only], disease or symptom duration, medication, and surgical history).

### Procedures

Patients had MRE and ultrasound in addition to any other enteric imaging or endoscopic investigations done during their usual clinical care.

MRE was done according to local standard clinical protocols (including the choice of oral contrast agent) on either 1·5 T or 3 T MRI platforms. We acquired a minimum dataset of sequences (appendix p 2). Ultrasound was done by radiologists or sonographers using standard platforms and both curvilinear and high-resolution probes, without oral or intravenous contrast agents (appendix p 3).

Across all sites, 28 practitioners interpreted the MRE and ultrasound studies (27 radiologists and 1 sonographer). Eight radiologists interpreted MRE only, three performed and interpreted ultrasound only, and 16 performed and interpreted ultrasound and interpreted MRE. All radiologists were affiliated with the British Society of Gastrointestinal and Abdominal Radiology, with declared subspecialty interest in gastrointestinal radiology, and had completed the Fellowship of the Royal College of Radiologists, with at least 1 year of subspecialty training in gastrointestinal radiology. The sonographer had received formal training according to their sites' local policies, was doing enteric ultrasound routinely, and had 20 years of experience. Radiologists interpreting MRE had a median of 10 years (IQR 6–11) of experience, and practitioners interpreting ultrasound had a median of 8 years (4–11) of experience. The median number of examinations done per month at each recruitment site during the conduct of the trial was 30 examinations (20–45) for MRE and 25 examinations (12–40) for ultrasound. Before trial commencement, we held a 2-day hands-on workshop for investigators to standardise ultrasound technique and agree on description of enteric findings.

MRE and ultrasound were interpreted by two different practitioners, each masked to the findings of the other, and masked to all other imaging, endoscopic, and clinical data except the group to which the patient was recruited (ie, newly diagnosed or relapse) and surgical history (since this information would normally be provided on clinical requests). Using case report forms, practitioners noted the presence and activity of Crohn's disease in the small bowel and colon, together with any extraenteric complications, using established criteria (appendix p 4).^6^, ^16^, ^25^ The segmental location of any disease was also recorded, using standard definitions;^24^ disease sites separated by more than 3 cm of normal bowel within a particular segment were recorded separately. Diagnostic confidence for disease presence was scored from 1 to 6, grouped into normal (levels 1–2), equivocal (levels 3–4), and abnormal (levels 5–6). A clinical report was then generated as per usual clinical practice.

Members of the local research team collected the findings of all other small bowel imaging or endoscopies done as part of usual care. These tests were done and interpreted according to usual clinical practice at local sites, without masking. A case report form recorded colonoscopic findings specifically.

For cases in which MRE and ultrasound had a discrepancy for the presence or location of small bowel disease, we did an arbiter small bowel investigation if patients had not already had additional small bowel imaging as part of usual care. We defined discrepancy as terminal ileal disease reported on either MRE or ultrasound in the absence of endoscopic visualisation, or disease reported in the small bowel upstream of the terminal ileum on either MRE or ultrasound. The nature of the additional test was left to local discretion and could include, for example, barium follow through, CTE, or capsule endoscopy. We also permitted repeat, targeted, and unmasked MRE or ultrasound to resolve discrepancies.

Where possible, we collected CRP concentration, calprotectin concentration, and the Harvey Bradshaw index at recruitment and repeated between 10 and 20 weeks later. We asked patients if they found MRE and ultrasound acceptable and which test attribute they considered to be the most important.

We used the construct reference standard model (panel diagnosis), incorporating the concept of clinical test validation—ie, whether test results are meaningful in practice.^23^ Specifically, we followed patients' clinical course for 6 months to assess the effect of MRE and ultrasound findings on clinical decision making and patient outcomes. Each recruitment site convened a series of consensus panels consisting of at least one local gastroenterologist and two radiologists (one local and one from another site); a histopathologist was available if required and a member of the trial management group attended to ensure uniformity of process. For each patient, the panel considered the images and results of all small bowel investigations (including MRE and ultrasound) and all additional information accrued over the follow-up period, including endoscopies, surgical findings, histopathology, Harvey Bradshaw index, CRP concentration, calprotectin concentration (and changes thereof), and clinical course. The panel recorded its opinion as to whether small bowel or colonic Crohn's disease was present, and, if so, whether disease was active. All panel decisions were recorded as present or absent, active or inactive, with no option of an indeterminate outcome. Disease could only be categorised as active if at least one objective marker was present (ulceration as seen at endoscopy, measured CRP concentration >8 mg/L, measured calprotectin concentration >250 μg/g, histopathological evidence of acute inflammation based on a biopsy sample or surgery within 2 months of trial imaging).

### Outcomes

The primary outcome was the per-patient difference in sensitivity between MRE and ultrasound for correct identification and localisation of small bowel Crohn's disease, irrespective of activity—ie, the extent of small bowel disease. To be truly positive for disease extent, the index test had to correctly locate the presence and segmental location of disease (terminal ileum, ileum, jejunum, or duodenum). Secondary outcomes reported here were specificity for disease extent, sensitivity and specificity for small bowel disease presence, the difference in per patient sensitivity and specificity for colonic disease presence and extent, and identification of active disease and comparative patient experience. Secondary outcomes also included comparative impact of MRE and ultrasound on clinician diagnostic confidence for presence of Crohn's disease and their influence on management, cost-effectiveness of MRE and ultrasound (compared to each other), diagnostic impact of novel MRE sequences (eg, diffusion-weighted imaging), influence of sequence selection on MRE diagnostic accuracy, diagnostic accuracy of small intestine contrast enhanced ultrasonography (SICUS) compared with standard ultrasound, influence of oral contrast agent and ingested volume on small bowel distension and patient experience during MRE, and interobserver variation, which will be reported elsewhere.

We reported most outcomes for the newly diagnosed and suspected luminal relapse groups individually, and for the terminal ileum and colon using colonoscopy as a standalone reference standard (when available) because of its robustness for identifying disease.

We prespecified all outcomes in the protocol^24^ except accuracy for individual small bowel segments (duodenum, jejunum, ileum), accuracy for disease presence and extent in the colon, and per-patient disease activity (small bowel and colonic disease combined), which were exploratory.

Safety reporting was limited to any suspected unexpected serious adverse reaction directly related to MRE, ultrasound, or any arbiter small bowel imaging test. Expected adverse reactions, such as contrast agent allergy, were not collated.

### Statistical analysis

We estimated that a sample size of 210 patients with small bowel disease would give 90% power to detect a clinically significant (10%) sensitivity difference for small bowel disease extent between MRE (83%, based on a sensitivity of 93% for disease presence and 90% for disease location) and ultrasound (73%, based on a sensitivity of 88% for disease presence and 83% for disease location), assuming 68% positivity for both tests and using methods for comparative studies.^24^, ^26^ A 10% difference in sensitivity was deemed to be clinically meaningful in routine practice by the study investigators at the time of trial design. We assumed a 70% prevalence of small bowel disease and 10% loss to follow-up or diagnosis with a disease other than Crohn's disease, which gave a target sample size of 334 patients across both groups (167 in the newly diagnosed group and 167 in the relapse group). The trial was not powered to detect differences between the groups, or between bowel segments.

We treated disease reported as equivocal as positive in the analysis. We calculated the primary outcome per patient. We based the secondary outcomes for bowel segments on all segments, excluding those resected at baseline (neoterminal ileum was considered as the terminal ileum).

We calculated a direct comparison of sensitivity and specificity differences between MRE and ultrasound using bivariate, multilevel, patient-specific (conditional), random-effects models, from paired data using meqrlogit in STATA 14.2 (College Station, TX, USA). When models did not converge due to small numbers of patients, we used McNemar's comparison of paired proportions to obtain univariable estimates and we calculated exact 95% CI. We did analysis by segment using a population-averaged, random-effects model (using logit, including robust standard errors). We based statistical significance on 95% CI.

This trial is registered with the International Standard Randomised Controlled Trial, number ISRCTN03982913.

### Role of the funding source

The funder (the National Institute for Health Research) stipulated a diagnostic accuracy trial using a cohort design but were not involved in the collection, analysis, or interpretation of data, or in the writing or submitting of this report. The corresponding author had full access to all data and final responsibility for the decision to submit for publication.

## Results

**Figure 1:**
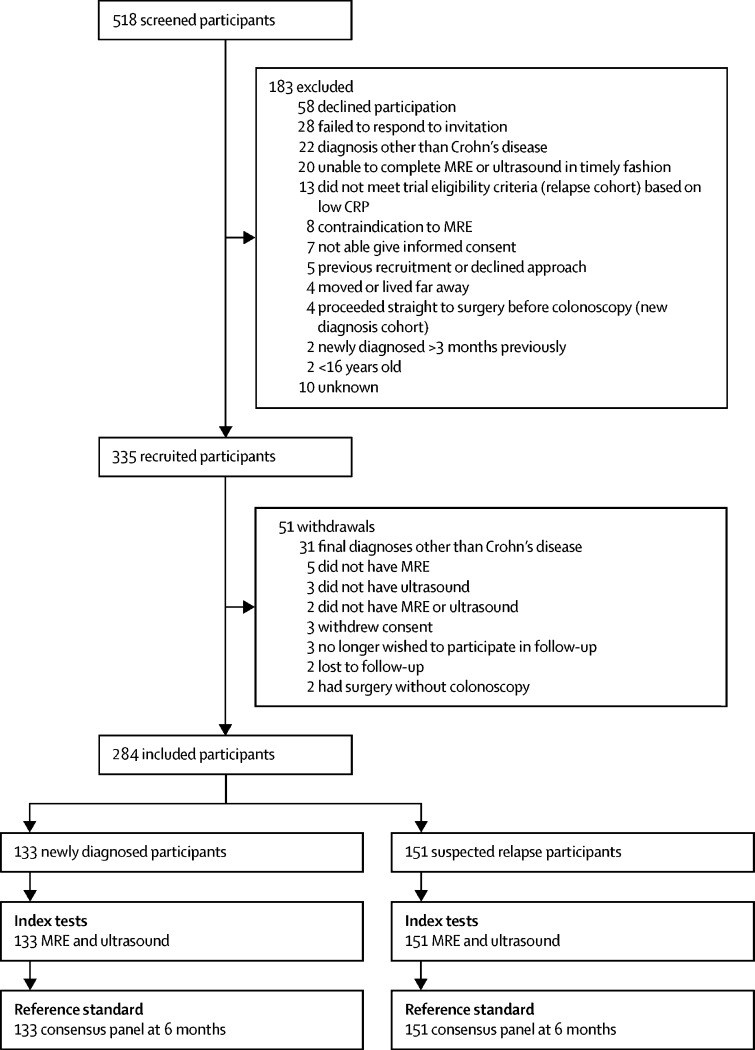
Trial profile CRP=C-reactive protein. MRE=magnetic resonance enterography.

We commenced recruitment on Dec 4, 2013, and completed it on Sept 30, 2016. Overall, we assessed 518 patients for eligibility, of whom 183 were excluded (figure 1). Of the 335 patients who entered the trial, 51 were subsequently excluded (20 men, median 30 years [IQR 24–41]); 31 did not have Crohn's disease, two were lost to follow-up, ten did not have MRE or ultrasound or both, six withdrew consent or no longer wished to participate in follow-up, and two newly diagnosed patients had surgery without colonoscopy. With a final group of 284, 133 were included in the newly diagnosed group and 151 in the relapse group (figure 1; table 1), including 154 (54%) women. Based on the reference standard, 233 (82%) of 284 patients had small bowel Crohn's disease (thereby meeting sample size stipulations), which was active in 209 (90%) patients (table 2). 129 (45%) of 284 patients had colonic disease, which was active in 126 (98%) patients. No data were missing for per-patient diagnosis of disease presence or disease extent, for the reference standard, MRE, or ultrasound.

**Table 1:** Patient demographics

		**New diagnosis (n=133)**	**Relapse (n=151)**
Sex			
	Male	69 (52%)	61 (40%)
	Female	64 (48%)	90 (60%)
Age (years)			
	16–25	49 (37%)	46 (30%)
	26–35	32 (24%)	36 (24%)
	36–45	18 (14%)	28 (19%)
	>45	34 (26%)	41 (27%)
Disease duration (years)			
	<1	NA	5 (3%)
	1–5	NA	45 (30%)
	6–10	NA	39 (26%)
	>10	NA	62 (41%)
Disease location (Montreal classification)*			
	L1	NA	56 (37%)
	L2	NA	17 (11%)
	L3	NA	74 (49%)
	L4	NA	4 (3%)
Disease behaviour (Montreal classification)*			
	B1	NA	80 (53%)
	B1p	NA	4 (3%)
	B2	NA	52 (34%)
	B2p	NA	1 (1%)
	B3	NA	12 (8%)
	B3p	NA	2 (1%)
Medication†			
	None	62 (47%)	32 (21%)
	Mesalazine	21 (16%)	26 (17%)
	Steroids	48 (36%)	28 (19%)
	Immunomodulators	16 (12%)	75 (50%)
	Anti-TNF antibodies	5 (4%)	42 (28%)
Previous enteric resection		1 (1%)‡	72 (48%)

Data are n (%). TNF=tumour necrosis factor. NA=not applicable.

* Montreal classification not collected for patients in the new diagnosis group.

† Patients could take more than one type of medication.

‡ Surgical resection for inflammatory mass 1 year before Crohn's disease diagnosis.

**Table 2:** Disease presence and activity based on the consensus reference standard

	**New diagnosis (n=133)**	**Suspected relapse (n=151)**	**Full cohort (n=284)**
**Disease presence**			
Small bowel disease present	111 (83%)	122 (81%)	233 (82%)
Colonic disease present	77 (58%)	52 (34%)	129 (45%)
Isolated small bowel disease present	56 (42%)	85 (56%)	141 (50%)
Isolated colonic disease present	22 (17%)	15 (10%)	37 (13%)
Both small bowel and colonic disease present	55 (41%)	37 (25%)	92 (32%)
Total number of patients with disease present	133 (100%)	137 (91%)	270 (95%)
Median number of involved small bowel segments, median (IQR), maximum	1 (1–1), 4	1 (1–1), 3	1 (1–1), 4
Median number of involved colonic segments, median (IQR), maximum	1 (0–3), 6	0 (0–1), 6	0 (0–2), 6
**Disease activity**			
Small bowel disease active	104 (94%)	105 (86%)	209 (90%)
Colonic disease active	76 (99%)	50 (96%)	126 (98%)
Total number of patients with disease active	130 (98%)	121 (88%)	251 (93%)
Criteria for activity*			
Ulceration at endoscopy	71 (55%)	26 (21%)	97 (39%)
CRP >8 mg/L	47 (36%)	57 (47%)	104 (41%)
Calprotectin >250 μg/g	41 (32%)	43 (36%)	84 (33%)
Histological evidence of activity	100 (77%)	36 (30%)	136 (54%)

Data are n (%), unless otherwise specified. CRP=C-reactive protein.

* Patients could meet more than one criteria for disease activity.

In 53 patients (24 from the newly diagnosed group and 29 from the relapse group), MRE and ultrasound were discrepant for small bowel disease presence or location, of whom 48 (91%) patients had an additional small bowel imaging test available to the consensus panel. The range of imaging, endoscopic, and biochemical data available to the consensus panels is shown in the appendix (p 5).

The sensitivity of MRE for the extent of small bowel disease (ie, presence and correct segmental location) was 80% (95% CI 72–86) compared with 70% (62–78) for ultrasound, a significant difference of 10% (1–18; p=0·027; table 3; appendix p 6). The specificity of MRE for the extent of small bowel disease was also significantly greater (95% [85–98]) than that of ultrasound (81% [64–91]), with a difference of 14% (1–27; p=0·039).

**Table 3:** Per-patient sensitivity and specificity for disease presence and extent against the consensus reference standard for patient groups combined

	**Sensitivity**					**Specificity**				
	Number of disease positive*	MRE	Ultrasound	Difference	p value	Number disease negative*	MRE	Ultrasound	Difference	p value
Small bowel disease extent†	233	80% (72 to 86)	70% (62 to 78)	10% (1 to 18)	0·027	51	95% (85 to 98)	81% (64 to 91)	14% (1 to 27)	0·039
Small bowel disease presence	233	97% (91 to 99)	92% (84 to 96)	5% (1 to 9)	0·025	51	96% (86 to 99)	84% (65 to 94)	12% (0 to 25)	0·054
Colonic disease extent†	129	22% (14 to 32)	17% (10 to 27)	5% (−5 to 15)	0·332	155	93% (87 to 97)	93% (87 to 97)	0% (−5 to 5)	1·000
Colonic disease presence	129	64% (50 to 75)	73% (59 to 83)	−9% (−23 to 5)	0·202	155	96% (90 to 98)	96% (90 to 98)	0% (−3 to 3)	1·000
Small bowel and colonic disease extent†	270	44% (36 to 54)	29% (21 to 38)	16% (6 to 25)	0·002	14	80% (42 to 96)	61% (23 to 89)	19% (−20 to 59)	0·337
Small bowel and colonic disease presence‡	270	78% (70 to 85)	71% (62 to 79)	7% (−2 to 15)	0·117	14	80% (42 to 96)	61% (23 to 89)	19% (−20 to 59)	0·335

Data are n, % (95% CI), or p value. MRE=magnetic resonance enterography.

* Patients by consensus reference standard.

† Agreement with reference standard for disease presence and segmental location.

‡ Agreement with reference standard for disease presence (patients with disease in the small bowel, colon, or both).

**Figure 2:**
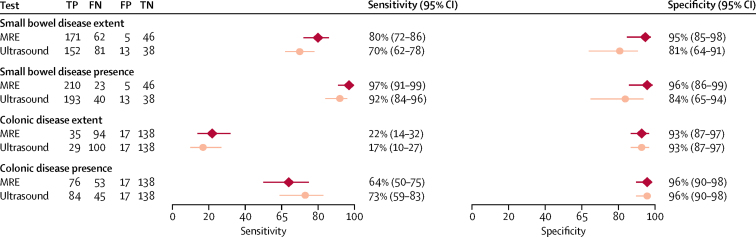
Sensitivity and specificity of MRE and ultrasound for the extent and presence of small bowel and colonic disease against the consensus reference standard FN=false negative. FP=false positive. MRE=magnetic resonance enterography. TN=true negative. TP=true positive. Error bars represent 95% CI.

The sensitivity of MRE (97% [95% CI 91–99]) for the presence of small bowel disease, regardless of location, was significantly greater than that of ultrasound (92% [84–96]), with a difference of 5% (1–9; table 3; figure 2).

The sensitivities of MRE and ultrasound for small bowel disease presence and extent in the newly diagnosed group and relapsed group were very similar to those estimated across all patients (table 4). However, ultrasound (67% [95% CI 49–81]) had significantly greater sensitivity for colonic disease presence than MRE (47% [31–64]) in the newly diagnosed patient group, with a difference of 20% (1–39). MRE and ultrasound had no significant difference in sensitivity or specificity for the extent and presence of colonic disease (table 3; figure 2). For both tests, sensitivity for colonic disease tended to be higher in the relapsed group than the newly diagnosed group (table 4), although the estimated sensitivity for colonic disease extent was poor for both groups.

**Table 4:** Sensitivity and specificity for disease presence and extent against the consensus reference standard according to patient cohort

	**New diagnosis group (n=133)**							**Suspected relapse group (n=151)**						
	Disease positive, disease negative*	Sensitivity			Specificity			Disease positive, disease negative*	Sensitivity			Specificity		
		MRE	Ultrasound	Difference	MRE	Ultrasound	Difference		MRE	Ultrasound	Difference	MRE	Ultrasound	Difference
Small bowel disease extent†	111, 22	77% (66 to 86)	66% (54 to 77)	11% (−2 to 24)	98% (82 to 100)	88% (64 to 97)	10% (−5 to 24)	122, 29	82% (72 to 89)	74% (62 to 83)	8% (−3 to 19)	92% (74 to 98)	75% (50 to 90)	17% (−3 to 37)
Small bowel disease presence	111, 22	96% (89 to 99)	92% (82 to 96)	4% (−1 to 10)	99% (84 to 100)	91% (65 to 98)	8% (−5 to 21)	122, 29	97% (91 to 99)	92% (82 to 96)	5% (0 to 11)	94% (76 to 99)	78% (50 to 92)	16% (−4 to 36)
Colonic disease extent†	77, 56	17% (9 to 30)	9% (4 to 19)	8% (−2 to 19)	93% (82 to 98)	92% (80 to 97)	1% (−7 to 10)	52, 99	31% (17 to 48)	33% (19 to 51)	−2% (−22 to 17)	93% (85 to 97)	94% (86 to 97)	−1% (−7 to 5)
Colonic disease presence	77, 56	47% (31 to 64)	67% (49 to 81)	−20% (−39 to −1)	96% (86 to 99)	95% (84 to 98)	1% (−5 to 7)	52, 99	84% (67 to 94)	80% (61 to 91)	4% (−11 to 20)	96% (88 to 98)	95% (89 to 99)	−1% (−5 to 4)
Small bowel and colonic disease extent†	133, 0	33% (22 to 46)	20% (12 to 30)	13% (1 to 26)	NA	NA	NA	137, 14	56% (43 to 68)	40% (28 to 52)	16% (2 to 31)	80% (42 to 96)	61% (24 to 88)	19% (−20 to 59)
Small bowel and colonic disease presence‡	133, 0	65% (52 to 76)	66% (53 to 77)	−1% (−15 to 13)	NA	NA	NA	137, 14	88% (79 to 93)	76% (64 to 85)	12% (2 to 22)	80% (42 to 96)	61% (23 to 89)	19% (−20 to 59)

Data are n or % (95% CI), unless otherwise specified. MRE=magnetic resonance enterography. NA=not applicable.

* Disease positive and disease negative patients by consensus reference standard.

† Agreement with reference standard for disease presence and segmental location.

‡ Agreement with reference standard for disease presence (patients with disease in the small bowel, colon or both).

The detection rate for individual small bowel and colonic segments is given in the appendix (p 7). Although the trial was not powered to detect differences on a segmental level, MRE was significantly more sensitive than ultrasound for ileal (84% [95% CI 67–93] *vs* 56% [38–73]) and rectal disease (44% [32–58] *vs* 22% [13–35]).

The sensitivity of MRE for active small bowel disease was 96% (95% CI 92–99) compared with 90% (82–95) for ultrasound, a significant difference of 6% (2–11; table 5). The specificity for active small bowel disease and for active colonic disease were not significantly different between tests (table 5). The sensitivity and specificity for active disease split by patient group were very similar to those estimated across all patients (appendix p 8).

**Table 5:** Per-patient sensitivity and specificity for the presence of active disease versus the consensus reference standard for patient groups combined

	**Sensitivity**					**Specificity**				
	Patients with active disease*	MRE	Ultrasound	Difference	p value	Patients with inactive disease*	MRE	Ultrasound	Difference	p value
Active small bowel disease†	209	96% (92 to 99)	90% (82 to 95)	6% (2 to 11)	0·010	75	83% (68 to 92)	77% (60 to 88)	6% (−8 to 20)	0·376
Active colonic disease†	126	63% (48 to 76)	66% (51 to 79)	−3% (−18 to 13)	0·735	158	97% (91 to 99)	98% (94 to 99)	−1% (−4 to 1)	0·304
Active small bowel and colonic disease‡	251	77% (68 to 85)	66% (56 to 75)	11% (1 to 21)	0·024	33	28% (10 to 56)	28% (10 to 56)	0% (−26 to 26)	1·000

Data are n, % (95% CI), or p value. MRE=magnetic resonance enterography.

* Patients by consensus reference standard.

† Agreement with reference standard for disease active.

‡ Agreement with reference standard for active disease presence (patients with disease in the small bowel, colon, or both).

21 patients had enteric fistulae, and seven patients had intra-abdominal abscess. MRE detected five (71%) of seven abscesses, whereas ultrasound detected three (43%) of seven abscesses. MRE detected 18 (86%) of 21 patients with enteric fistulae compared with 11 (52%) of 21 patients for ultrasound.

Against a colonoscopic standard of reference (available in 186 patients), MRE had a sensitivity of 97% (95% CI 91–99) for terminal ileal disease presence compared with a sensitivity of 91% (79–97) for ultrasound, a difference of 6% (−1 to 12; appendix p 9). The sensitivity for colonic disease presence was modest for both MRE (41% [26–58]) and ultrasound (49% [33–65]) and was not statistically different.

Of responding patients, 128 (88%) of 145 patients rated MRE as acceptable and 144 (99%) of 146 patients rated ultrasound as acceptable. Diagnostic accuracy was rated as the most important test attribute.

No serious adverse events or any other adverse events were reported.

## Discussion

In the METRIC trial, we found that both MRE and ultrasound were highly accurate for detecting small bowel Crohn's disease, achieving 97% sensitivity for MRE and 92% sensitivity for ultrasound. Barium fluoroscopy has long been advocated as a sensitive test for mucosal disease inaccessible to endoscopy, although its support is limited to a handful of small studies^2^ and its accuracy is increasingly questioned.^27^ Conversely, against a rigorous ileocolonoscopic reference standard, we found that MRE and ultrasound achieved 97% and 91% sensitivity for terminal ileal disease, strongly supporting their transition to first-line investigations, and positioning them as competitive and viable diagnostic alternatives to invasive ileocolonoscopy. Of the two, we found MRE had significantly higher sensitivity and specificity than ultrasound for small bowel extent, and higher sensitivity for disease presence. Overall, no significant difference was found in diagnostic accuracy for colonic disease (consistently lower than for small bowel disease), although ultrasound had greater sensitivity than MRE in newly diagnosed patients. To our knowledge, the METRIC trial is the largest prospective multicentre trial to date directly comparing diagnostic accuracy of MRE and ultrasound for the presence, extent, and activity of Crohn's disease in the same patients.

Our primary outcome combined those aspects necessary to stage small bowel Crohn's disease correctly—ie, is disease present, and, if so, where? Both presence and extent dictate subsequent therapeutic strategy. For example, the finding of additional proximal small bowel disease might tip the balance towards medical rather than surgical intervention in the face of otherwise isolated terminal ileal disease. As expected, sensitivity for disease extent was lower than that for disease detection alone.

Our detection rates were at the upper end of estimates from previous meta-analyses.^6^, ^9^, ^10^, ^11^, ^12^, ^13^, ^14^, ^15^, ^16^, ^17^, ^18^, ^19^, ^20^ Dong and colleagues^12^ estimated ultrasound to have a sensitivity 88% and a specificity of 97%; Liu and colleagues^17^ reported corresponding figures of 86% sensitivity and 93% specificity for MRE. However, the primary literature is markedly heterogeneous, which affects the validity of point estimates. Most studies were single centre and typically recruited fewer than 50 patients, and many were methodologically poor.^17^, ^21^ Direct comparison of diagnostic tests in the same patients is advocated as the optimal method for diagnostic accuracy studies^22^ because differences are attributable directly to the tests and not to differences between participants or study methods. Such head-to-head comparisons are rare in the medical literature.^15^ Reference standards might also be applied inconsistently, with endoscopy, surgery, and imaging all variably employed. For example, in a comparative study with ultrasound, Castiglione and colleagues^28^ used MRE without any additional reference standard in many recruits, which introduces the potential for incorporation bias.

We used the construct reference standard model (panel diagnosis), which incorporates multiple data sources with clinical outcome.^23^ Although such an approach does have limitations, including potential panel bias, it is considered a very robust method for diagnostic accuracy studies in which a single external reference standard is elusive.^23^ To reduce incorporation bias, patients without supplementary small bowel imaging had a third small bowel investigation whenever discrepancy between MRE and ultrasound arose. Notably, when our analysis was limited to an ileocolonoscopic reference standard, any differences in accuracy between MRE and ultrasound closely mirrored those found using the consensus panel reference.

We recruited approximately equally from two patient groups: newly diagnosed Crohn's disease and established disease with relapse. Both groups are clinically distinct and important, and might manifest with differing disease phenotypes; prevalence of stricturing and penetrating disease increases with time.^29^ Noting that the METRIC trial was not powered to detect differences between these two patient groups, we found that sensitivity for small bowel disease was similar, although specificity tended to be lower in patients in the relapse group. Conversely, sensitivity for colonic disease was higher in the relapse group, but was still poor for colonic disease extent (about 30%).

In newly diagnosed patients, ultrasound achieved significantly greater sensitivity for colonic disease than MRE (67% *vs* 47%). Optimised colonic assessment with MRE requires purgation and fluid distension,^30^ which are both omitted from routine MRE protocols; however, ultrasound generally relies on assessing the manually compressed uncleansed colon wall. Accuracy for both techniques in the colon still falls short of colonoscopy, and accuracy with MRE is somewhat lower than previously reported.^31^, ^32^ By way of explanation, ileocolonoscopy and histopathology results were available to the consensus reference panel for most patients (particularly those newly diagnosed) and are exquisitely sensitive for early mucosal disease, beyond the resolution of cross-sectional imaging. Our outcomes were dependent on disease presence regardless of severity. Previous single-centre explanatory studies either use groups enriched with more advanced colonic disease,^32^ or report sensitivity for deep rather than superficial mucosal disease.^31^

Most patients found MRE and ultrasound acceptable, although slightly more found ultrasound acceptable. This outcome is perhaps expected given the different attributes of the two tests. However, patients rated diagnostic accuracy as the most important test attribute, consistent with previous work,^33^ suggesting patients will tolerate greater discomfort for improved test performance.

The METRIC trial does have some limitations. It was conceived as a large pragmatic trial^34^ since the medical literature is replete with small explanatory studies. We recruited from a range of hospital settings, both teaching and district general, and used local imaging protocols to enhance generalisability. The 28 practitioners all declared a specialist interest in gastrointestinal radiology and were representative of those reporting NHS small bowel imaging in terms of training and experience. We specifically avoided using a small number of highly experienced practitioners since they would not represent a national workforce. However, we acknowledge that specialist practitioners working in high volume practices might achieve sensitivities in excess of our findings. Imaging was interpreted according to local clinical practice to mirror real-world procedures within the NHS and enhance generalisability of our results. We acknowledge that masking practitioners to individual patient history does not mirror usual clinical practice, but this precaution was necessary to isolate diagnostic test accuracy as far as possible. We cannot, however, exclude occasional inadvertent unmasking of reporting practitioners. Recruited patients were representative of those having MRE and ultrasound in daily practice, although we did exclude pregnant women, patients having routine therapeutic response assessment, and patients with contraindications to MRI. Our results are therefore highly likely to be extrapolable across the NHS and similar health-care settings. The prevalence of active disease was predictably high given our recruited patient groups. Therefore, the reported high specificity of MRE and ultrasound should be viewed in this context.

We did not standardise the third small bowel investigation whenever discrepancy between MRE and ultrasound arose, and this decision was left to the discretion of the recruitment site. Direct mucosal visualisation is possible with push enteroscopy^35^ or capsule endoscopy,^36^ but to insist on such investigations was not practicable in the setting of a pragmatic multicentre trial given their cost, relatively inadequate availability, and probable negative effect on patient compliance and safety. Push enteroscopy, for example, is a highly invasive and specialised investigation, and attracts a small but well documented risk of major complications, such as perforation.^37^ Similarly the risk of capsule retention is around 8% in patients with known Crohn's disease^38^ and specificity is questioned.^39^ We also considered that the invasive nature of capsule endoscopy or enteroscopy would result in considerable spectrum bias relating to differences between patients who would and would not agree to consent (even if they were available and affordable).

To reduce incorporation bias from MRE or ultrasound, we required at least one independent biochemical, endoscopic, or histological marker of disease activity before a patient could be diagnosed with active small bowel or colonic disease. Biochemical markers, such as calprotectin and CRP concentrations, provide evidence at the patient level, but the reference standard consensus panel also had access to a range of additional clinical material when making their decision, including endoscopy and a range of small bowel imaging investigations.

Some data suggest that the diagnostic accuracy of ultrasound can be improved with an oral contrast load (SICUS), particularly for luminal stenosis, and intravenous contrast enhanced ultrasound (CEUS) might have utility for assessing disease activity.^40^ However, neither SICUS nor CEUS have disseminated as first-line investigations outside specialist units, and if used are often employed as problem solving tools.^40^, ^41^ Standard ultrasound is overwhelmingly the most commonly used technique in routine clinical practice. Future prospective research could consider inclusion of SICUS and CEUS in trial design.

Diagnostic accuracy is clearly paramount when patients are investigated, but interobserver variability and cost-effectiveness are also of great importance and will be reported elsewhere, together with a more detailed consideration of patient experience.

In summary, we found that both ultrasound and MRE achieve excellent diagnostic accuracy for the extent and activity of small bowel Crohn's disease in newly diagnosed patients and those who have relapsed, and both tests are valid firstline investigations. In an NHS setting, MRE is generally the preferred radiological investigation when available because its sensitivity and specificity exceed ultrasound significantly.
