# Supplementary material for: Diagnostic accuracy of magnetic resonance enterography and small bowel ultrasound for the extent and activity of newly diagnosed and relapsed Crohn's disease (METRIC): a multicentre trial
Source: Lancet Gastroenterol Hepatol. 2018 Jun 18;3(8):548–58. doi: 10.1016/S2468-1253(18)30161-4 (PMC6278907; doi:10.1016/S2468-1253(18)30161-4)
Supplement: Supplementary appendix [file mmc1.pdf]

THE LANCET
Gastroenterology & Hepatology

Supplementary appendix

This appendix formed part of the original submission and has been peer reviewed. We post it as supplied by the authors.

Supplement to: Taylor S A, Mallett S, Bhatnagar G, et al. Diagnostic accuracy of magnetic resonance enterography and small bowel ultrasound for the extent and activity of newly diagnosed and relapsed Crohn's disease (METRIC): a multicentre trial. *Lancet Gastroenterol Hepatol* 2018; published online June 15. http://dx.doi.org/10.1016/S2468-1253(18)30161-4.

Web Appendix

Table 1. Recruitment sites and recruitment totals

Recruitment site	Principle Investigator	Total patients screened	Total patients recruited (new diagnosis) [n (%) of total]	Total patients recruited (suspected relapse) [n (%) of total]	Total patients recruited (both patient cohorts [n (%) of total]	Total patients withdrawn [n (%) of total]	Total patients in final cohort (new diagnosis) [n (%) of total]	Total patients in final cohort (suspected relapse) [n (%) of total]	Total patients in final cohort (both cohorts) [n (%) of total]
University College London Hospital	Professor Stuart Taylor	177	66 (39)	69 (41)	135 (40)	19 (36)	52 (39)	64 (42)	116 (41)
Queen Alexandra Hospital, Portsmouth	Dr Tony Higginson	66	32 (19)	27 (16)	59 (18)	9 (18)	28 (20)	22 (15)	50 (18)
St James's University Hospital, Leeds Teaching Hospitals, NHS Trust, Leeds	Dr Damian Tolan	69	29 (17)	22 (13)	51 (15)	4 (8)	27 (20)	20 (13)	47 (17)
Ninewells Hospital, Dundee	Dr Ian Zealley	71	11 (6)	15 (9)	26 (8)	3 (6)	9 (7)	14 (9)	23 (7)
St Marks Hospital, Harrow	Dr Arun Gupta	78	8 (5)	16 (10)	24 (7)	4 (8)	5 (4)	15 (10)	20 (6)
Radcliffe Hospital, Oxford	Dr Andrew Slater	39	15 (9)	11 (7)	26 (8)	7 (14)	9 (7)	10 (7)	19 (7)
St Georges Hospital, London	Dr Richard Pollok	11	6 (4)	5 (3)	11 (3)	4 (8)	2 (2)	5 (3)	7 (3)
Royal Free Hospital, London	Dr Peter Wylie	7	1 (1)	2 (1)	3 (1)	1 (2)	1 (1)	1 (1)	2 (1)
TOTAL		518	168	167	335	51	133	151	284

Table 2a. MRE sequence protocol

Minimum	Optional
Coronal steady state free precession gradient echo (SSFP GE) sequences without fat saturation	Axial steady state free precession gradient echo (SSFP GE) sequences without fat saturation
Hyoscine butylbromide 20mg IV	Axial fast spin echo (FSE) T2W sequence with fat saturation
Axial and coronal fast spin echo (FSE) T2W sequences without fat saturation	Axial contrast-enhanced coronal T1W sequences with fat saturation (60-70 sec post injection)
Coronal coronal fast spin echo (FSE) T2W sequence with fat saturation	Coronal steady state free precession gradient echo (SSFP GE) dynamic Motility sequences
Axial diffusion weighted images (b values 50 and 600)	
Non-enhanced coronal T1W sequence with fat saturation followed by contrast-enhanced coronal T1W sequences with fat saturation (60-70 sec post injection)	

Table 2b. US sequence protocol

Minimum	Optional
Curved (convex) probe (2-5Mhz)	2 cups of water 10 mins before to distend the duodenum
Linear probe >5Mhz	
Colour Doppler (typical setting 6-9m/s)	

Table 3. Suggested protocol imaging signs of active disease

MRE	US
Wall thickening, increased mural T2 signal, increased mesenteric T2 signal, increased enhancement (mucosal or layered), ulceration or abscess,	Wall thickening, focal hyperechoic mesentery (with or without fat wrap), isolated thickened submucosal layer, poorly defined anti-mesenteric border, increased doppler vascular pattern, ulceration or abscess

Table 4. Investigations and results available to the consensus panels

	New diagnosis [n (%)]<br>N=133	Relapse [n (%)]<br>N=151
MR enterography	133 (100)	151 (100)
US	133 (100)	151 (100)[a]
Colonoscopy	123 (92)	66 (44)[b]
Gastroscopy	11 (8)	6 (4)
Sigmoidoscopy	5 (4)	12 (8)
Capsule endoscopy	10 (8)	8 (5)
CT enterography	4 (3)	9 (6)
CT abdo pelvis	21 (16)	13 (9)
MR enteroclysis	4 (3)	6 (4)
MRI abdomen and/or pelvis	5 (4)	8 (5)
Barium FT	8 (6)	19 (13)
Barium enteroclysis	3 (2)	7 (5)
Hydrosonography	28 (21)	36 (24)
White cell scan	0 (0)	0 (0)
CRP (baseline)	127 (95)	145 (96)
HBI (baseline	124 (93)	142 (94)
Calprotectin (baseline)	87 (65)	89 (59)
CRP (10-20 weeks)	108 (81)	120 (79)
HBI (10-20 weeks)	71 (53)	77 (51)
Calprotectin (10-20 weeks)	53 (40)	65 (43)
Surgical resection specimen (post recruitment)	1 (1)	2 (1)
Other	8 (6)	20 (13)

[a] One patient with hydrosonography was used as main US scan

[b] Three patients with follow-up colonoscopy, no data available to consensus panel

Table 5. Cross tabulation of MRE and US against the reference standard for small bowel disease extent. Both patient cohorts combined.

A. Patients with small bowel disease by the reference standard

Comparison of correct small bowel extent		USS		
		+	-	Total
**MRE**	+	125	46	171
	-	27	35	62
	Total	152	81	233

Trial outcomes were calculated from bivariate multilevel patient specific (conditional) random effects models, not directly from 2x2 table

B. Patients without small bowel disease by the reference standard

Comparison of correct small bowel extent		USS		
		-	+	Total
**MRE**	-	35	11	46
	+	3	2	5
	Total	38	13	51

Trial outcomes were calculated from bivariate multilevel patient specific (conditional) random effects models, not directly from 2x2 table

Supplementary Table 6. Per segment sensitivity and specificity for disease presence against the consensus reference standard.

	Sensitivity % (CI 95%)				Specificity % (CI 95%)			
	**Number of disease positive[a]**	**MRE**	**US**	**Difference (P value)**	**Number of negative[a]**	**MRE**	**US**	**Difference**
**Small bowel segments**								
Duodenum[b]	8	25 (7 to 59)	25 (7 to 59)	0 (-13 to 13)	276	100 (99 to 100)	99 (97 to 100)	1 (0 to 3)
Jejunum	13	71 (38 to 91)	63 (32 to 86)	8 (-29 to 46)	271	99 (93 to 100)	99 (94 to 100)	0 (-2 to 1)
Ileum	38	84 (67 to 93)	56 (38 to 73)	28 (8 to 49)	246	93 (87 to 97)	93 (87 to 96)	0 (-4 to 4)
Terminal ileum	217	96 (91 to 99)	92 (84 to 96)	4 (0 to 8)	67	97 (90 to 99)	93 (82 to 98)	4 (-2 to 10)
**Colonic segments[c]**								
Caecum	78	46 (35 to 57)	46 (35 to 57)	0 (-12 to 12)	147	96 (92 to 99)	90 (85 to 94)	6 (0 to 12)
Ascending	67	49 (38 to 61)	49 (38 to 61)	0 (-10 to 10)	200	96 (93 to 98)	92 (88 to 95)	4 (0 to 8)
Transverse	61	46 (34 to 58)	44 (32 to 57)	2 (-12 to 15)	218	97 (93 to 98)	95 (91 to 97)	2 (-1 to 5)
Descending	59	53 (40 to 65)	41 (29 to 54)	12 (-1 to 24)	221	98 (95 to 99)	95 (91 to 97)	3 (0 to 6)
Sigmoid	76	46 (35 to 57)	43 (33 to 55)	3 (-11 to 16)	203	96 (92 to 98)	93 (89 to 96)	3 (-1 to 7)
Rectum	54	44 (32 to 58)	22 (13 to 35)	22 (9 to 35)	228	97 (94 to 99)	93 (89 to 96)	4 (0 to 7)

[a] Segments by consensus reference standard
[b] McNemar's test due to small number of patients with disease
[c] Analysis for individual colonic segments uses a population average approach to compare accuracy for individual colon segment

Table 7. Per patient sensitivity and specificity for the presence of active disease against the consensus reference standard, according to patient cohort.

	New diagnosis N=133							Suspected relapse N=151						
		Sensitivity % (CI 95%)			Specificity % (CI 95%)				Sensitivity % (CI 95%)			Specificity % (CI 95%)		
	DA,DI[a]	MRE	US	Difference (P value)	MRE	US	Difference (P value)	DA,DI[a]	MRE	US	Difference (P value)	MRE	US	Difference (P value)
Active Small bowel disease[b]	104,29	96 (90 to 99)	90 (79 to 96)	6 (0 to 13)	90 (68 to 98)	83 (56 to 95)	7 (-11 to 25)	105,46	96 (90 to 99)	90 (79 to 96)	6 (0 to 13)	79 (57 to 91)	73 (51 to 88)	6 (-14 to 25)
Active Colonic disease[b]	76,57	48 (30 to 66)	55 (36 to 72)	-7 (-28 to 14)	96 (88 to 99)	97 (90 to 99)	-1 (-5 to 4)	50,101	83 (63 to 93)	81 (59 to 92)	2 (-14 to 19)	96 (89 to 99)	98 (93 to 99)	-2 (-5 to 2)
Active Small bowel and colonic disease[c]	130,3	64 (50 to 77)	59 (44 to 72)	5 (-10 to 20)	0 (0 to 56)	0 (0 to 56)	0 (-33 to 33)	121,30	88 (78 to 94)	73 (59 to 84)	15 (3 to 26)	40 (25 to 58)	40 (25 to 58)	0 (-22 to 22)

[a]Disease active (DA), disease inactive (DI) patients by consensus reference standard

[b] Agreement with reference standard for disease activity

[c] Agreement with reference standard for active disease presence (patients with disease in small bowel, colon or both). McNemar's test for specificity due to small number of new patients with inactive disease

Table 8. Sensitivity and specificity for terminal ileal and colonic disease presence and against an ileo colonoscopy reference. Both patient cohorts combined.

	Sensitivity % (CI 95%)				Specificity % (CI 95%)			
	Number of disease positive[a]	MRE	US	Difference (P value)	Number of disease negative[a]	MRE	US	Difference (P value)
Terminal ileum	105	97 (91 to 99)	91 (79 to 97)	6 (-1 to 12)	81	41 (21 to 64)	33 (15 to 57)	8 (-14 to 30)
Colonic disease extent[b]	109	3 (1 to 11)	2 (0 to 8)	1 (-2 to 4)	77	94 (81 to 98)	89 (73 to 96)	5 (-3 to 14)
Colonic disease presence	109	41 (26 to 58)	49 (33 to 65)	-8 (-26 to 9)	77	95 (85 to 98)	90 (76 to 96)	5 (-3 to 13)
Colonic segments[c]								
Caecum	73	22 (14 to 33)	25 (16 to 36)	-3 (-14 to 9)	101	72 (63 to 80)	65 (56 to 74)	7 (0 to 13)
Ascending	62	26 (16 to 38)	23 (14 to 35)	3 (-6 to 12)	121	88 (80 to 92)	81 (73 to 87)	7 (0 to 13)
Transverse	54	24 (15 to 37)	24 (15 to 37)	0 (-9 to 9)	132	92 (86 to 96)	90 (84 to 94)	2 (-2 to 6)
Descending	58	27 (18 to 40)	24 (15 to 37)	3 (-6 to 13)	128	95 (90 to 98)	93 (87 to 96)	2 (-1 to 6)
Sigmoid	74	24 (16 to 35)	28 (19 to 40)	-4 (-17 to 9)	111	94 (87 to 97)	94 (87 to 97)	0 (-6 to 6)
Rectum	61	26 (17 to 39)	13 (7 to 24)	13 (2 to 25)	125	97 (92 to 99)	94 (88 to 97)	3 (-2 to 8)

[a] Patients or colonic segments by ileo colonoscopy reference standard

[b] Agreement with reference standard for disease presence and segmental location

[c] Analysis for individual colonic segments uses a population average approach to compare imaging accuracy for individual colon segment

Metric Trial investigators

Jade Dyer, Pranitha Veeramalla, Sue Tebbs, Steve Hibbert (Comprehensive Clinical Trials Unit at University College London (UCL), Institute of Clinical Trials and Methodology, London); Richard Ellis, Fergus Thursby-Pelham, Richard Beable, Nicola Gibbons, Claire Ward (Portsmouth Hospitals NHS Trust, Portsmouth); Anthony O'Connor, Hannah Lambie, Rachel Hyland, Nigel Scott, Roger Lapham, Doris Quartey, Deborah Scrimshaw (St James's University Hospital, Leeds Teaching Hospitals NHS Trust, Leeds); Helen Bungay, Maggie Betts, Simona Fourie (Oxford University Hospitals NHS Trust, Oxford); Niall Power (Royal Free London NHS Foundation Trust, London); Rajapandian Ilangovan, Uday Patel, Evgenia Mainta, Phillip Lung, Ian Johnston, Mani Naghibi, Morgan Moorghen, Adriana Martinez, Francois Porte (St Mark's Hospital, London North West University Healthcare NHS Trust, Harrow); Christopher Alexakis, James Pilcher, Anisur Rahman, Jonny Vlahos, Rebecca Greenhalgh, Anita Wale (St George's Hospital, London); Teresita Beeston, Wivijin Piga, Joey Clemente, Farooq Rahman, Simona de Caro, Shameer Mehta, Roser Vega, Roman Jastrub, Harbir Sidhu, Hameed Rafiee (University College Hospital, London); Mairead Tennent, Caron Innes, Craig Mowat, Gillian Duncan (Ninewells Hospital, Dundee); and Steve Morris (UCL, London).
